# Autonomic Nervous System Regulation Effects of Epipharyngeal Abrasive Therapy for Myalgic Encephalomyelitis/Chronic Fatigue Syndrome Associated With Chronic Epipharyngitis

**DOI:** 10.7759/cureus.33777

**Published:** 2023-01-14

**Authors:** Ito Hirobumi

**Affiliations:** 1 Otolaryngology, Ito ENT Clinic, Funabashi, JPN

**Keywords:** heart rate, chronic fatigue syndrome, myalgic encephalomyelitis, myalgic encephalomyelitis/chronic fatigue syndrome (cfs), epipharyngeal abrasive therapy (eat), autonomic nervous system regulation, reilly phenomenon, vagus nerve reflex, heart rate variability analysis

## Abstract

Objective: To evaluate the autonomic nerve stimulation effect of epipharyngeal abrasive therapy (EAT) on myalgic encephalomyelitis/chronic fatigue syndrome (CFS) associated with chronic epipharyngitis. Heart rate variability analysis was performed. The study was conducted by analyzing heart rate variability.

Subjects and methods: A total of 29 patients with chronic epipharyngitis who underwent EAT from July 2017 to April 2018 were classified into two groups: 11 patients in the CFS group and 18 patients in the control group without CFS. The patients were classified as phase 1 during bed rest, phase 2 during nasal endoscopy, phase 3 during nasal abrasion, and phase 4 during oral abrasion. Electrocardiographic recordings were made, and autonomic function was compared and evaluated by measuring heart rate, coefficient of variation on R-R interval (CVRR), coefficient of component variance high frequency (ccvHF), and low frequency/ccvHF ratio (L/H) for each of the four phases. The Shapiro-Wilk test was performed to confirm the normality of the two groups, and the parametric test was selected. A repeated measures analysis of variance was performed to assess changes over time between the four events in the two groups. Multiple comparisons were corrected by the Bonferroni method. Comparisons between resting data and three events within each group were performed by paired t-test.

Results: The CFS group had an increased baseline heart rate compared to the control group, and the CFS group had a greater increase in parasympathetic activity and a decrease in heart rate with nasal abrasion. Oral abrasion elicited a pharyngeal reflex and increased heart rate and both sympathetic and parasympathetic activity.

Conclusion: The CFS group was in a state of dysautonomia due to autonomic overstimulation, with an elevated baseline heart rate. The CFS group was considered to be in a state of impaired autonomic homeostasis, with an increased likelihood that overstimulation would induce a pathological vagal reflex and the Reilly phenomenon would develop. The direct effects of EAT on the autonomic nervous system were considered to be vagus nerve stimulation and the regulation of autonomic function by opposing stimulation input to sympathetic and parasympathetic nerves. As an indirect effect, bleeding from the epipharyngeal mucosa due to abrasion was thought to restore the function of the cerebral venous and lymphatic excretory systems and the autonomic nerve center.

## Introduction

Various theories have been reported on the etiology of myalgic encephalomyelitis/chronic fatigue syndrome (CFS), including viral infection, endocrine abnormalities, immune abnormalities, metabolic abnormalities, and autonomic nervous system dysfunction. The various abnormalities seen in CFS are thought to form a cascade in relation to each other [1], and the complaints in CFS are thought to be based on abnormalities in brain function [2].

Hotta et al. reported three mechanisms of action of epipharyngeal abrasive therapy (EAT) [3-6]. The three mechanisms are (1) direct mucocutaneous astringent, bactericidal, and anti-inflammatory effects of zinc chloride; (2) improvement of local circulation by phlebotomy; and (3) vagus nerve stimulation (VNS). EAT has been used as a treatment for chronic epipharyngitis [7]. EAT has also been reported to be effective as a treatment for the sequelae of novel coronavirus disease (COVID) infection (long COVID (LC)) [8-10] and CFS [11], but its mechanism of action is still largely unresolved.

Tracey et al. reported that VNS has an inflammatory reflex that suppresses inflammation via the immune system [12]. VNS, which applies the inflammatory reflex, has been used as a treatment for intractable epilepsy, autoimmune diseases, intractable headaches, and depression [5]. EAT is expected to have the same effects as VNS. However, stimulation of parasympathetic nerves may induce pathological vagal reflexes [13], and chronic persistent stimulation of sympathetic nerves or inflammation-related factors may induce pathological autonomic reflexes, resulting in decreased autonomic nervous system activity and autonomic overstimulation syndrome (Reilly phenomenon) [14]. These pathological vagal reflexes and the Reilly phenomenon may be responsible for the variety of symptoms caused by chronic epipharyngitis. Thus, it can be inferred that EAT may have both a therapeutic effect and a potential for exacerbating chronic epipharyngitis, two potentially conflicting effects.

The purpose of this study was to evaluate the efficacy of EAT in CFS associated with chronic epipharyngitis, to clarify the pathogenesis of CFS, and to elucidate the mechanism of action of EAT on CFS. Heart rate (HR) variability analysis is a noninvasive method to measure and evaluate autonomic function.

Ito reported that the mechanism of the effect of EAT on the autonomic nervous system was examined using HR variability analysis [15]. In this study, we discuss the two conflicting mechanisms of action of EAT on the autonomic nervous system, and report the results of this study, considering that one of the therapeutic effects of EAT may be its autonomic modulatory effect.

## Materials and methods

A patient who visited our hospital with chief complaints of postnasal drip, hoarseness, abnormal feeling in the throat, sore throat, etc. was diagnosed with chronic epipharyngitis according to Tanaka's diagnostic criteria [4]. When patients complain of sore throat or dysphagia without any particular findings, a differential diagnosis is made with consideration of diseases such as elongation of the styloid process (Eagle syndrome) [16]. From July 2017 to April 2018, 288 patients with chronic epipharyngitis were examined, and 29 patients underwent EAT while monitoring the electrocardiogram: total mean age = 39.8 ± 17.1 years; five males (mean age = 26.2 ± 17.5 years); 24 females (mean age = 42.6 ± 15.9 years); and male-to-female ratio = 1:4.8.

Eleven patients (33.7 ± 15.1; three males and eight females) with chief complaints of chronic fatigue and autonomic neuropathy at the initial visit were classified as the CFS group. Eighteen patients (mean age 43.5 ± 17.6; two males and 17 females) who did not complain of other autonomic symptoms were classified as the control group, and electrocardiographic recordings were compared between the two groups. Oral and written informed consent was obtained from all patients, and the study was conducted in compliance with the Declaration of Helsinki. When handling the data and other materials related to the study, we took great care to protect the confidentiality of the subjects, and we did not include any information that could identify the subjects when publishing the results of the study. This is a retrospective observational study based on existing medical record information, and no new samples or information were obtained. The study was approved by the Ethics Committee of the Chiba Prefecture Health Physicians Association (approval no.: 20210601008).

Diagnosis and treatment were performed using a band-limited optical endoscope system (Pentax EPK-i7000 Video Processor and VNL11-J10 Video Scope with a 3.5 mm diameter outer tip, PENTAX Medical, Tokyo, Japan). After the patient was placed at rest, he was anesthetized with 1% Xylocaine as a nasal procedure to prevent pain in the nasal cavity, and hyperemia and swelling of the nasal mucosa were removed with a 0.01% solution of adrenaline. After confirming that the ECG was stabilized, an endoscope was inserted nasally and a series of treatments with EAT was initiated. First, an endoscopic diagnosis was made, and then epipharyngeal abrasion was performed nasally using a Rouze swab moistened with 1% zinc chloride solution while observing the epipharynx. Next, epipharyngeal abrasion was performed orally using a Zhermack pharyngeal crimp cotton swab soaked in 1% zinc chloride solution. After observation of bleeding, the endoscope was removed and the EAT was terminated.

The ECG recordings were classified into the following four phases: (1) resting and waiting (phase 1); (2) nasally inserting an endoscope for examination (phase 2); (3) nasal abrasion treatment of the epipharyngeal mucosa (phase 3); and (4) oral abrasion treatment of the epipharyngeal mucosa (phase 4). HR variability analysis was performed for each of the four phases. HR variability analysis was performed using HR variability analysis software (Reflex Meijin, Crosswell Co., Ltd., Yokohama, Japan). HR is affected by autonomic nervous activity involved in respiration and systemic circulation, and HR variability is observed in the ECG R-R interval. High-frequency components (HFC) and low-frequency components (LFC) are common indices of fluctuations in the cardiovascular system. The coefficient of variation on the R-R interval (CVRR) is an aggregate of components (coefficient of component variance high frequency (ccvHF), low frequency (LF), etc.) of frequency analysis results and is used as the sum of autonomic nervous activity [17]. ccvHF is used as an index of parasympathetic function [18]. The LF component reflects sympathetic and parasympathetic functions, and the LF/ccvHF ratio (L/H) divided by ccvHF is used as an index of sympathetic function. Measurements were taken within the interval of each event, with a 30-second ECG recording interval as one data length, and the R-R interval (detected by the peak interval of the R wave) for each beat within that interval was sampled at a sampling frequency of 1000 Hz and the average value was calculated. HFC and LFC were defined as 0.15-0.40 Hz and 0.04-0.15 Hz, respectively. Four items (HR, CVRR, ccvHF, and L/H) were evaluated in the four phases and statistically examined.

An F-test confirmed that the two groups (CFS group and control group) had equal variances. A Shapiro-Wilk test was performed for each endpoint to ensure normal distribution and a parametric test was selected. A Smirnov-Grubbs test was performed on each endpoint measure and no outliers were identified. A repeated measures analysis of variance (rm ANOVA) was performed to assess changes over time between the four events. Multiple comparisons were corrected with the Bonferroni method. The statistical analysis was performed by rm ANOVA for changes over time in phases 1-4, and comparisons were made among the four events. Multiple comparisons were corrected by the Bonferroni method. The comparison between phase 2, phase 3, and phase 4 of the CFS group and the control group was performed using a paired t-test, with phase 1 at rest before the start of EAT as the reference value. The mean value of phase 1 of each group was used as the reference value, and the difference between the mean values of phase 2, phase 3, and phase 4 was used to determine the rate of change for the comparison. The statistical software EZR version 2.6-2 was used for statistical analysis. A difference with a risk rate of less than 5% was considered statistically significant.

## Results

The 288 patients included 65 males, with a mean age of 43.8 ± 18.5 years, and 223 females, with a mean age of 45 ± 15.1 years. The mean overall age was 44.7 ± 16.0 years, with a sex ratio of 1:3.5. Table 1 presents a summary of the chief complaints of all patients. The CFS group consisted of 11 patients (3.8%), and the control group consisted of 18 patients (Table 1).

**Table 1:** Summary of principal complaints The following is a breakdown of the chief complaints of the 288 patients seen. Chronic fatigue syndrome was reported in 11 cases (3.8%).

Principal complaint	Number of cases	%
Posterior rhinorrhea	130	45.1
Hoarseness	42	14.6
Abnormal pharyngeal sensation	41	14.2
Sore throat	22	7.6
Dizziness	13	4.5
Cough	12	4.2
Chronic fatigue syndrome	11	3.8
Headache	10	3.5
Ear closure	4	1.9
Atopic dermatitis	4	1.9
IgA nephropathy	1	0.3
Pustulosis palmoplantaris	1	0.3
Rheumatoid arthritis	1	0.3
Sleep apnea syndrome	1	0.3
Total number of cases	288	100

**Figure 1:**
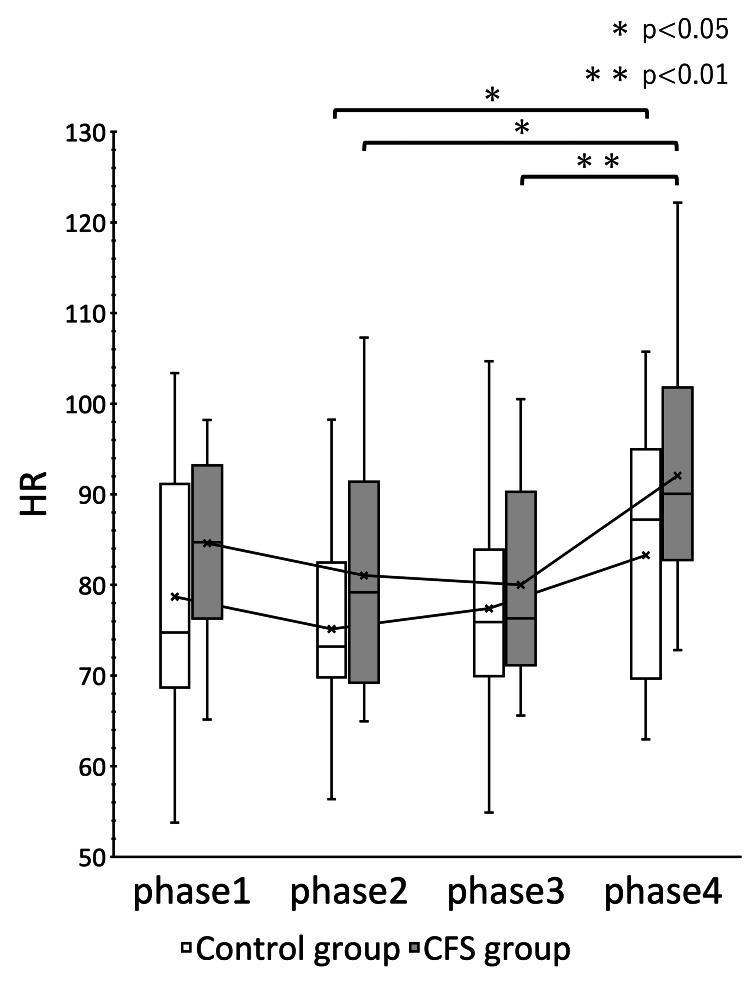
Change in HR over time The white bars represent the control group and the gray bars represent the CFS group. The vertical axis shows HR in bpm. The abscissa shows the measured values of phases 1-4, indicating the time course. Measurements are shown in the box and whisker diagram. The center line indicates the median value, and the X mark indicates the mean value. The mean of each group is connected by a solid line. rm ANOVA showed that both the CFS group and the control group were significantly different (p < 0.01). Multiple comparisons showed that the CFS group was significantly different between phase 2 and phase 4 (p = 0.044) and between phase 3 and phase 4 (p < 0.01), while the control group was significantly different between phase 2 and phase 4 (p = 0.011). HR: heart rate; CFS: chronic fatigue syndrome; rm ANOVA: repeated measures analysis of variance.

**Figure 2:**
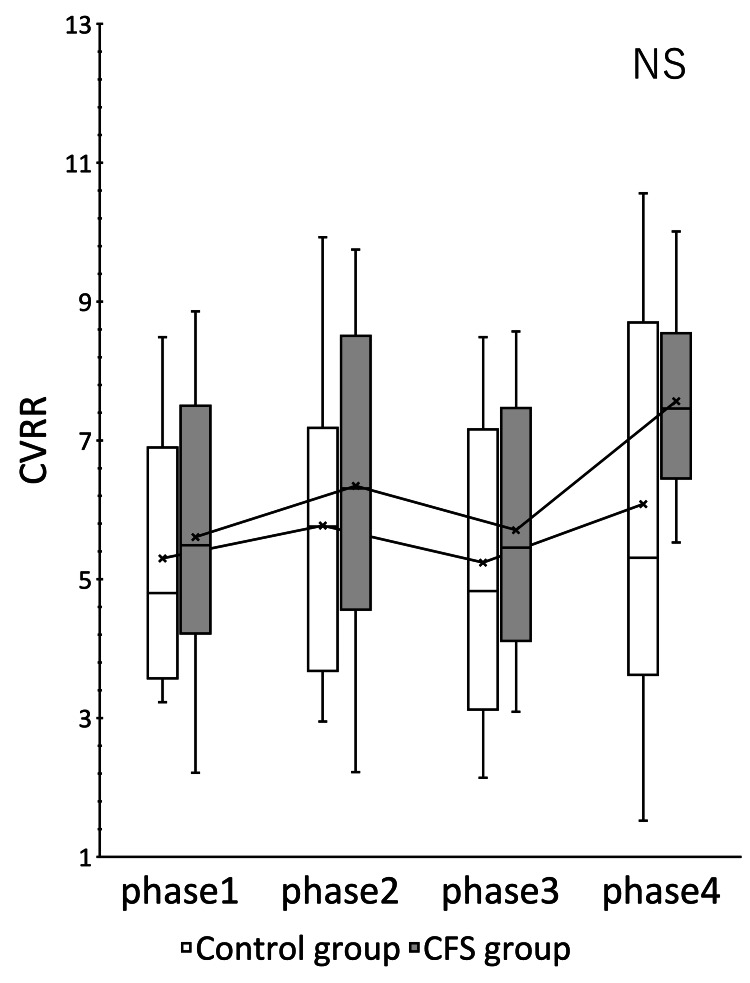
Change in CVRR over time The vertical axis indicates CVRR, expressed in %. The rm ANOVA showed a significant difference in the CFS group (p = 0.012). Multiple comparisons showed that the CFS group showed a near-significant change between phase 1 and phase 4 (p = 0.052). The control group showed no significant difference. CVRR: coefficient of variation on the R-R interval; CFS: chronic fatigue syndrome; rm ANOVA: repeated measures analysis of variance.

**Figure 3:**
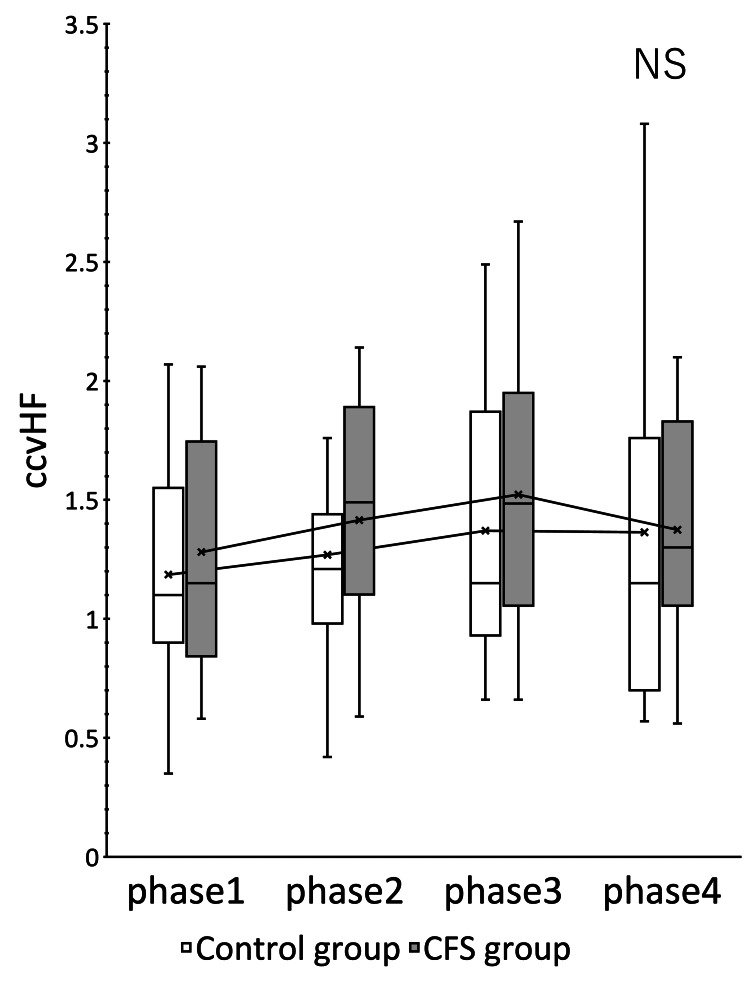
ccvHF change over time The vertical axis indicates ccvHF, expressed in %. The rm ANOVA showed that the CFS group was close to significant (p = 0.077), but not significantly different from the CFS group. ccvHF: coefficient of component variance high frequency; CFS: chronic fatigue syndrome; rm ANOVA: repeated measures analysis of variance.

**Figure 4:**
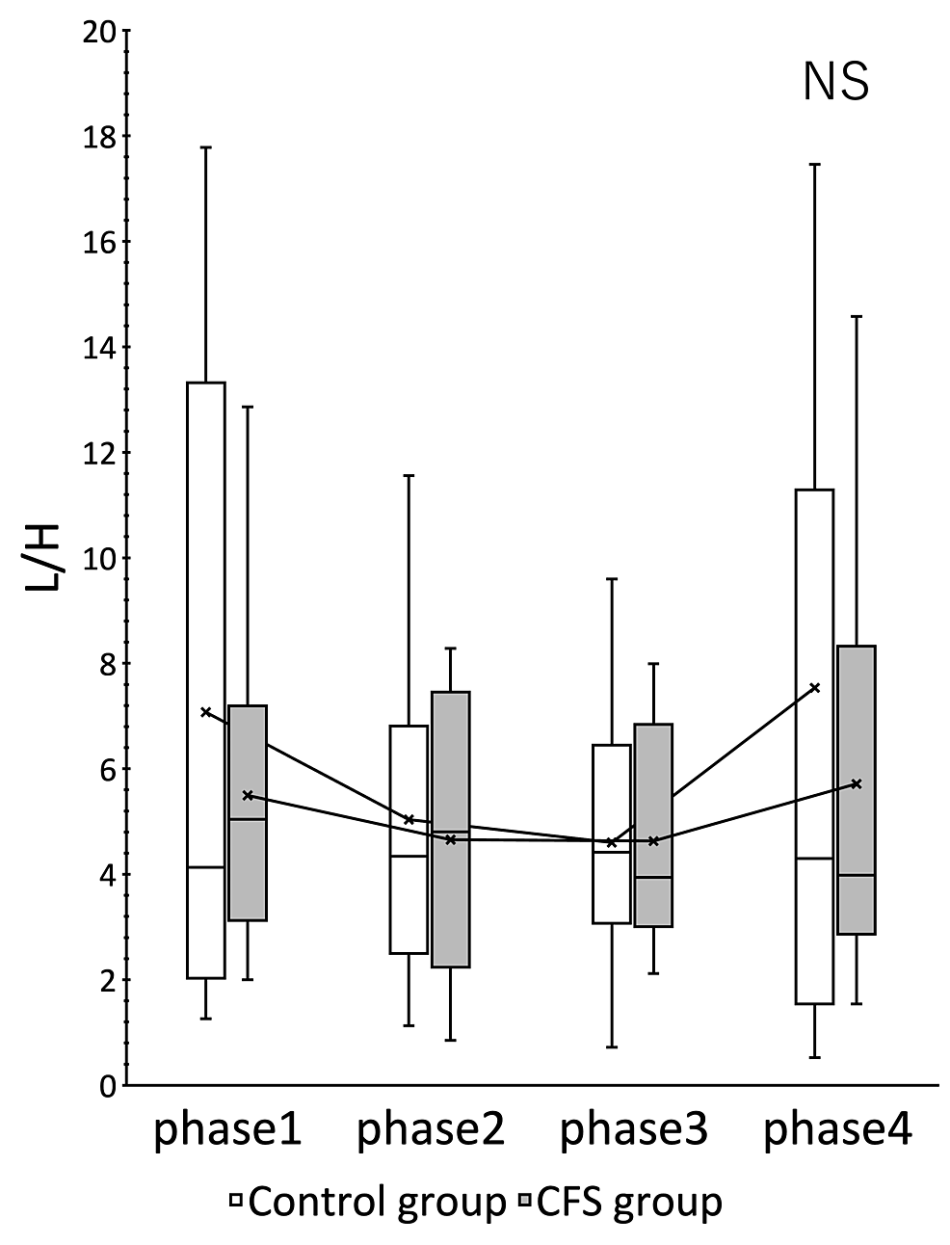
Change in L/H over time The vertical axis indicates L/H, and the units are ratios. L/H showed a decreasing trend in phase 3 and an increasing trend in phase 4. CFS: chronic fatigue syndrome; L/H: low frequency/ccvHF ratio.

The CFS group showed significant changes in HR (p = 0.000) and CVRR (p = 0.012) (Figures 1, 2). ccvHF was close to significant with p = 0.077 (Figure 3). L/H showed no significant difference but showed a decreasing trend with increasing ccvHF in phase 3 and an increasing trend with decreasing ccvHF in phase 4 (Figure 4). In multiple comparisons, HR was significantly different between phases 2 and 4 (p = 0.044), and between phases 3 and 4 (p = 0.009). CVRR was close to significant between phases 1 and 4 (p = 0.052). In the control group, there was no significant change in HR (p = 0.000). The control group showed a significant change in HR (p = 0.000). In multiple comparisons, HR was significantly different between phase 2 and phase 4 (p = 0.011), and CVRR was close to significantly different (p = 0.056).

Within the same group, a paired t-test comparison between the two groups at rest and between the three phases is shown (Table 2). The CFS group showed significant changes in HR in phase 3, CVRR in phase 4, and ccvHF in phase 3. A comparison of the mean HR in phase 1 between the control group and the CFS group showed that the resting baseline HR of the CFS group was 5.3% higher than that of the control group. The CFS group had a higher HR in phase 2 and phase 4, but the CFS group had a lower HR in phase 3. The variability of HR and CVRR in phase 2 did not differ from that of the control group, while ccvHF and L/H were more variable in the CFS group. The variability of ccvHF in phase 3 was higher in the CFS group (22.6%) than in the control group (11.1%). The variability of L/H in phase 3 showed a decrease of 14.2% in the CFS group versus a large decrease of 30.0% in the control group. The variability of CVRR in phase 4 showed an increase of 17.4% in the control group versus a large increase of 35.6% in the CFS group.

**Table 2:** Measurements, percent change, and p-values for each phase of the CFS group (n = 11) and the control group (n = 18) Measurements are shown as mean ± standard deviation. Percentage change is shown as %. Within each group, two-arm comparisons between phase 1 and the other three phases (i.e., phase 1 vs. phase 2, phase 1 vs. phase 3, and phase 1 vs. phase 4) were performed by paired t-test. The CFS group showed significant changes in HR in phase 3, CVRR in phase 4, and ccvHF in phase 3. The control group showed significant changes in HR in phase 2 and phase 4. The resting baseline heart rate in phase 1 was higher in the CFS group, but the reduction in HR in phase 3 was greater in the CFS group. CVRR showed a greater rate of increase in phase 4 in the CFS group. L/H tended to be more variable in the control group. HR: heart rate; CFS: chronic fatigue syndrome; CVRR: coefficient of variation on the R-R interval; ccvHF: coefficient of component variance high frequency; L/H: low frequency/ccvHF ratio.

Item	Unit	Group	Phase 1	Phase 2	％	P-value	Phase 3	％	P-value	Phase 4	％	P-value
HR	bpm	CSF group	83.82 ± 10.84	79.96 ± 12.58	-4.6	0.063	78.44 ± 10.72	-6.4	0.040	89.33 ± 11.15	6.6	0.098
		Control group	79.59 ± 13.16	76.21 ± 11.46	-4.2	0.010	78.69 ± 12.79	-1.1	0.497	85.74 ± 15.96	7.7	0.045
CVRR	%	CSF group	5.62 ± 2.12	6.37 ± 2.51	13.3	0.335	5.76 ± 1.93	2.5	0.719	7.62 ± 2.37	35.6	0.009
		Control group	5.12 ± 1.76	5.89 ± 1.93	15.0	0.062	5.00 ± 2.06	-2.4	0.674	6.01 ± 2.80	17.4	0.106
ccvHF	%	CSF group	1.24 ± 0.51	1.39 ± 0.52	12.1	0.111	1.52 ± 0.64	22.6	0.030	1.33 ± 0.50	7.3	0.492
		Control group	1.16 ± 0.46	1.22 ± 0.47	4.3	0.504	1.30 ± 0.61	11.1	0.173	1.33 ± 0.71	12.0	0.152
L/H	Ratio	CSF group	5.74 ± 3.01	4.65 ± 2.77	-16.5	0.371	4.78 ± 2.06	-14.2	0.295	6.09 ± 4.33	5.9	0.838
		Control group	6.96 ± 5.59	6.51 ± 5.27	-6.5	0.771	4.87 ± 2.70	-30.0	0.085	8.08 ± 8.95	16.1	0.483

## Discussion

The purpose of this study was to analyze the pathogenesis of CFS with chronic epipharyngitis and to elucidate the mechanism of action of EAT. Subjects were divided into two groups (CFS group and control group), and EAT was performed. CVRR showed a significant change over time in the CFS group. This indicates that the CFS group showed greater variability in response to EAT stimulation compared to the control group.

First, the mean age of the 288 patients with chronic epipharyngitis in this study was 44.7 years, with a male-to-female ratio of 1:3.5. Ohno reported that the mean age of chronic epipharyngitis cases was 42.9 years, with a male-to-female ratio of 1:3 [19]. The results of this study suggest that chronic epipharyngitis is a common disease among middle-aged women. The frequency of chief complaints among the examinees in this study was as follows: posterior rhinorrhea in 45.1%, hoarseness in 14.6%, dysphonia in 14.2%, sore throat in 7.6%, and CFS in 3.8%. Mogitate et al. reported the frequency of chief complaints among the patients seen as posterior rhinorrhea in 41.2%, sore throat in 11.8%, abnormal sensation in 10.8%, IgA nephropathy in 7.8%, and CFS in 7.8% [20]. The study population may reflect the average chronic epipharyngitis case. Since chronic epipharyngitis cases are often associated with CFS, it is considered important to evaluate autonomic function. HR variability analysis is a noninvasive method to evaluate autonomic function. We conducted this study because we believe that HR variability analysis may be a useful tool for elucidating the autonomic nerve-stimulating effects of EAT.

EAT has been reported to be similar to intranasal sphenopalatine ganglion stimulation (INSPGS). EAT stimulation is thought to have parasympathetic stimulating effects [9]. In the present study, an increase in parasympathetic activity during nasal abrasion with a concomitant decrease in HR was observed. Ito reported on the autonomic reflex activity induced by EAT stimulation using HR variability analysis. Ito reported HR variability analysis and autonomic reflex activity induced by EAT stimulation. During nasal abrasion and nasal endoscopy, autonomic activity was increased and CVRR showed bimodal changes in activity. During nasal abrasion, parasympathetic activity is stimulated and sympathetic activity is suppressed. During oral rubbing, both sympathetic and parasympathetic nerves are stimulated simultaneously, but sympathetic and parasympathetic nerves move oppositely, and there is a time lag in their activity. Therefore, it was reported that EAT stimulation has both sympathetic and parasympathetic stimulating effects, but the response depends on the timing and site of stimulation [15]. During nasal abrasion, parasympathetic activity was increased and HR was decreased, but the decrease in HR was greater in the CFS group than in the control group. It is known that stimuli such as mental stress, excessive pain, excretion, and abdominal visceral disease induce vagal reflexes such as hypotension, HR reduction, and syncope and that HR is reciprocally coupled with parasympathetic activity. EAT may induce vagal reflexes, but the CFS group showed a greater decrease in HR than the control group. The CFS group was more likely than the control group to have a vagal reflex induced by EAT.

Comparison within the same group showed that the CFS group had a higher baseline HR and a greater decrease in HR during nasal abrasion than the control group, while the CFS group had a greater increase in HR during oral abrasion. The CFS group also showed greater variability in ccvHF and CVRR. The CFS group showed greater variability in autonomic and parasympathetic activities induced by EAT stimulation, suggesting that the CFS group is more sensitive to stimulation. Kuratsune et al. reported that CFS patients exhibit both sympathetically dominant and parasympathetically depressed states [21]. Persistent inflammatory stimulation by chronic epipharyngitis stimulates the sympathetic reflex and exhausts the parasympathetic function. Such a persistent state induces a state of autonomic ataxia. As a result, the baseline HR is considered to be elevated in CFS with chronic epipharyngitis [22]. In the present study, an elevated baseline HR was also observed in the CFS group. This increase in baseline HR may indicate a state of decreased parasympathetic function. HR regulation by the sympathetic and parasympathetic nerves is also a phenomenon called accentuated antagonism, in which HR regulation by the parasympathetic nerves is enhanced in the presence of sympathetic activity [23]. The increased variability of HR in the CFS group may be due to the fact that the sympathetic nervous system is stimulated by the persistent inflammatory stimulus, and the sensitivity of the vagus nerve is also increased. In CFS caused by chronic epipharyngitis, persistent inflammatory stimulation stimulates the sympathetic reflex and exhausts the parasympathetic function, but the vagus nerve reflex is easily triggered.

It is known that overstimulation of the autonomic nervous system causes microcirculatory disturbances and hemorrhagic lesions due to ischemia and reperfusion disorders in various organs throughout the body, resulting in the Reilly phenomenon [14,24]. In the control group, the fluctuation of measured values is relatively small, suggesting that autonomic homeostasis is at work. In the CFS group, the fluctuation of each endpoint is large and autonomic homeostasis is considered to be impaired. The possibility that pathological vagal reflexes are triggered by overstimulation and that the Reilly phenomenon may occur is considered to be increased. The results of this study suggest that the CFS group is in a state of autonomic dysfunction due to autonomic overstimulation and that the vagus nerve reflex is easily triggered. Porges proposed the polyvagal theory, which states that the autonomic response to stress consists of a hierarchical regulatory response of two types of vagal nerves (ventral and dorsal vagal systems) and the sympathetic nervous system [25]. In the stress response, the ventral vagal system initially acts in an inhibitory manner and regulates the HR and other functions. When the sympathetic nervous system is stimulated, the ventral vagal system is released from its inhibitory role and a reflex response is triggered. One of the possible pathophysiologies of CFS is the inability of the vagus nervous system to suppress and regulate sympathetic over-reactivity in response to stress, i.e., vagal homeostasis may be impaired. CFS patients with persistent chronic stimulation have impaired autonomic regulation and elevated baseline HR. During nasal abrasion stimulation, the increase in parasympathetic activity and the associated decrease in HR are controlled by respiratory sinus arrhythmia (RSA) in the ventral vagal system [26], whereas, in CFS, the ventral vagal system is over-suppressed. In CFS, however, the ventral vagal system may be excessively suppressed, leading to the development of autonomic neuropathy and other complaints.

The direct autonomic nerve stimulating effect of EAT may be, first, to stimulate the parasympathetic function and restore the vagus nerve reflex. Second, by stimulating both sympathetic and parasympathetic nerves simultaneously to induce the pharyngeal reflex, it may work to normalize the onset of the pharyngeal reflex. However, the autonomic reflex activity by EAT may induce pathological vagal reflexes or Reilly's phenomenon because the response differs depending on the stimulus intensity, stimulus duration, stimulus site, and sensitivity of the individual.

Currently, the stellate ganglion block (SGB) is used as a treatment method to suppress or stimulate the autonomic nervous system. SGB temporarily blocks the sympathetic nervous system to increase parasympathetic activity. After SGB blocks nerve function, autonomic activity fluctuates due to a rebound phenomenon [27]. SGB repetitive stimulation therapy shakes the autonomic nervous system, regulates the autonomic nervous system balance of sympathetic and parasympathetic nerves, and activates self-repair functions [28]. It is speculated that the mechanism of the therapeutic effect of EAT on the autonomic nervous system is similar to that of SGB repetitive stimulation therapy and that the conflicting excitatory and inhibitory input stimuli from simultaneous stimulation of the sympathetic and parasympathetic nervous systems may induce the pharyngeal reflex to adjust and activate autonomic nervous system functions.

Tracey proposed an inflammatory protective system in the vagus nerve system and named it the inflammatory reflex [12]. VNS, which applies this mechanism, has been used as a treatment for autoimmune diseases and depression [29]. The results of this study revealed that EAT has vagus nerve stimulating effects, suggesting that EAT may express anti-inflammatory effects through the inflammatory reflex. EAT is considered to be a simple VNS treatment that is not invasive like SGB and does not require expensive mechanical devices or surgery like VNS. Although it is not possible to compare the superiority of EAT to SGB or VNS at this point, EAT may improve various clinical symptoms caused by chronic epipharyngitis through its anti-inflammatory effect and activation of autonomic nervous system activity. EAT may be effective against brain dysfunction in CFS, complaints due to autonomic dysfunction, and brain fog due to LC by activating autonomic nervous system activity.

Kuroiwa et al. reported that the hypothalamus and periventricular organs are important as security gates of the stress center [30], and Iliff et al. reported that cerebrospinal fluid dynamics are regulated by the glymphatic system, and the nasopharyngeal circulation pathway is important as a drainage route for cerebral waste products [31]. When the epipharyngeal mucosa becomes congested, circulatory disturbance of the stress center is caused, resulting in various autonomic symptoms. Uebaba reported that Batson's venous plexus is important in cerebral and spinal venous drainage. The vertebral venous plexus within the spinal canal is called Batson's venous plexus, and it communicates with the venous plexus outside the spinal canal to act as an excretory pathway. Since Batson's venous plexus has no valve structure, venous blood flow is slow and prone to stagnation. Also, the direction of flow is easily reversed. Venular congestion is likely to occur in chronic epipharyngitis, and venous congestion occurs in the vertebral venous plexus such as the axis and atlas when circulation disorder occurs in the epipharyngeal mucosa. It has been reported that this causes circulatory disturbance in the autonomic nerve center, resulting in autonomic dysfunction [32].

The pharyngeal veins and lymphatic vessels have an important function as cerebral veins and lymphatic drainage channels, and when the circulatory disturbance is induced by chronic epipharyngitis, the cerebral waste excretion system may be impaired, resulting in autonomic neuropathy. Directly, EAT has the function of improving inflammation of the local mucosa and resolving the autonomic nervous system stimulation state but it is also thought that physical abrasion and phlebotomy of the diseased epipharyngeal mucosa may restore the function of the cerebral venous and lymphatic excretory system and improve autonomic nervous system function in the brainstem, thalamus, and hypothalamus. Indirectly, EAT may also have an effect on stimulating autonomic nervous system function.

The present study was a retrospective observational study and no controlled trial was conducted using healthy subjects as controls. Further clarification of the differences between EAT-induced autonomic reflexes and pathological vagal reflexes or the Reilly phenomenon is needed, such as the degree of difference in stimulation intensity, stimulation duration, and stimulation site, as well as the difference in sensitivity among individuals. In addition, this study did not examine the prognosis of CFS patients treated with EAT. This is an issue for future studies.

## Conclusions

The post-hoc analysis comparing the CFS group with the control group showed that the CFS group was in a state of autonomic ataxia caused by autonomic nerve overstimulation and that the vagus nerve reflex was easily induced. The EAT nasal abrasion increased parasympathetic activity and decreased HR in CFS patients. Oral EAT rubbing induced the pharyngeal reflex and increased HR and autonomic nervous system activity.

The direct effects of EAT on the autonomic nervous system may be the VNS effect by parasympathetic stimulation and the autonomic regulation effect by opposing excitatory and inhibitory stimulus inputs to sympathetic and parasympathetic nerves. Indirect effects on the autonomic nervous system may include improvement of hypothalamic autonomic function through the recovery of cerebral venous and lymphatic excretory system function. These autonomic nerve-stimulating effects may act synergistically to produce the effect of EAT.
